# Safety of Endoscopy in Peritoneal Dialysis Patients

**DOI:** 10.14309/ctg.0000000000000379

**Published:** 2021-07-01

**Authors:** Joon Sung Kim, Eunha Jung, Sun Hyung Kang, Jeong-Seon Ji, Yu Kyung Cho, Bo-In Lee, Young-Seok Cho, Byung-Wook Kim, Hwang Choi, Hyun Yong Jeong, Myung-Gyu Choi, Jae Myung Park

**Affiliations:** 1 Division of Gastroenterology, Department of Internal Medicine, Incheon St. Mary's Hospital, College of Medicine, The Catholic University of Korea, Seoul, Korea;; 2 Division of Gastroenterology, Department of Internal Medicine, Seoul St. Mary's Hospital, College of Medicine, The Catholic University of Korea, Seoul, Korea;; 3 Division of Gastroenterology, Department of Internal Medicine, Chungnam National University School of Medicine, Daejeon, Korea.

## Abstract

Endoscopic procedures can provoke peritonitis in patients receiving peritoneal dialysis (PD). The aim of this study was to assess the development of peritonitis after endoscopic procedures in PD patients.

**METHODS:**

We retrospectively reviewed the data from PD patients who underwent endoscopies in 3 tertiary hospitals between 2008 and 2018. The patients were grouped into nonprophylactic, prophylactic, and prior antibiotic therapy groups. The incidence of peritonitis within 7 days of endoscopy was assessed. We also examined the factors associated with peritonitis.

**RESULTS:**

There were 1,316 endoscopies performed in 570 PD patients. The peritonitis rate after endoscopy was 3.0%. Specifically, the peritonitis rate was 1.8% for esophagogastroduodenoscopies, 4.2% for the colonoscopy group, and 5.3% for the sigmoidoscopy group. The prior antibiotic therapy group showed a significantly higher risk of peritonitis (odds ratio = 4.6; 95% confidence interval: 2.2–9.6; *P* < 0.01). Prophylactic antibiotics were not associated with reducing peritonitis. Therapeutic colonoscopies such as polypectomy were associated with an increased risk of developing peritonitis (odds ratio = 6.5; 95% confidence interval: 1.6–25.9). However, biopsies were not associated with an increased risk of peritonitis.

**DISCUSSION:**

Prophylactic antibiotics did not reduce the risk of peritonitis after endoscopy in PD patients. Therapeutic colonoscopies such as polypectomy and prior antibiotic therapy before endoscopy were associated with an increased risk of peritonitis.

## INTRODUCTION

Peritonitis is a common complication arising in peritoneal dialysis (PD) therapy and results in considerable morbidity and death (1). Several pathogenic mechanisms have been suggested, including intraluminal, periluminal, hematogenous, transmural, omental lymphogenous, and ascending routes (2). Endoscopic procedures play a critical role in the diagnosis and treatment of gastrointestinal (GI) diseases. Such procedures can increase the risk of peritonitis in PD patients by inflation and irritation of the bowel wall, leading to the translocation of microbes across the gut wall to other sites such as the peritoneum and the blood stream (2). Whether prophylactic antibiotics are universally needed before endoscopic procedures in PD patients is not clear. There have been case reports of peritonitis developing after esophagogastroduodenoscopies (EGD), colonoscopies, and colonoscopic polypectomies (3–6).

Generally, prophylactic antibiotics are recommended for invasive endoscopic procedures such as percutaneous endoscopic gastrostomy (7). The results of previous studies vary on the role of antibiotic prophylaxis for prevention of peritonitis and the invasiveness of endoscopic procedures such as polypectomy as a risk factor for peritonitis (8–11). The risk of bacteremia is considered to be low for routine upper and lower endoscopic procedures, and prophylactic antibiotics are generally not recommended. However, prophylactic antibiotics are recommended before colonoscopies in PD patients (2,7). Studies on the development of peritonitis after an endoscopy are limited in number and quality (8–11). Controversy exists on the role of antibiotic prophylaxis before endoscopy in PD patients.

The excessive prescribing of antibiotics may be associated with side effects and the development of antibiotic resistance. However, peritonitis is a grave event with serious consequences, which may justify the use of prophylactic antibiotics. The primary aim of our study was to assess the development of peritonitis and the role of prophylactic antibiotics after endoscopic procedures in PD patients. As a secondary objective, we examined the patient- and procedure-related characteristics associated with an increased risk of peritonitis in PD patients receiving an endoscopy.

## METHODS

### Study design

This was a retrospective study conducted at 3 tertiary hospitals in Korea. This study was approved by the institutional review board of each hospital. Patients were included if they (i) were 18 years or older, (ii) were receiving PD, and (iii) underwent endoscopic evaluation between January 2008 and December 2018. Patients with evidence of peritonitis before the endoscopic procedure were excluded. We extracted patient- and procedure-related data from electronic medical records. Some of the data were published previously in an article unrelated to the aim of the current manuscript (10).

### Patient characteristics

Demographic data, including age, sex, the duration of dialysis, the causes of renal failure, and history of peritonitis, were assessed. Laboratory data included albumin, blood urea nitrogen, creatinine, and potassium serum levels and white blood cell counts in whole blood. The use of proton-pump inhibitors and oral or intravenous antibiotics before the endoscopic procedures was also investigated. The American Society for GI Endoscopy guidelines recommend antibiotics such as ampicillin (1 g) plus a single dose of aminoglycoside, with or without metronidazole given intravenously immediately before an endoscopic procedure to reduce the risk of peritonitis (7). The use of antibiotics was further classified into 3 groups. The nonprophylactic group received no antibiotics before their endoscopic procedures. The prophylactic group received ampicillin (1 g) or cefazolin (1 g) with or without metronidazole given intravenously before the endoscopic procedures. The prior antibiotic therapy group consisted of patients who did not have any evidence of peritonitis but were receiving antibiotics for other infectious causes before the endoscopic procedure.

### Endoscopic procedure characteristics

Data regarding the dates, types, and indications for the endoscopic procedures as well as the endoscopic findings were investigated. The endoscopic procedures were classified into the EGD group, the colonoscopy (CS) group (colonoscopies with or without EGD), and the sigmoidoscopies (Sig) group (with or without EGD). We also determined whether the endoscopic procedures were diagnostic or invasive. Invasive procedures were further classified into the biopsy group and the therapeutic group (hemostasis and polypectomy).

### Outcomes

The primary outcome of this study was the development of peritonitis within 1 week of the endoscopic procedure. Peritonitis was diagnosed when abdominal pain and cloudy fluid occurred with or without fever and when the peritoneal fluid white blood cell count was >100/mm^3^ with >50% polymorphonucleocytes. PD effluent was sent for hematological and microbiological examination when the patients complained of abdominal pain or if their fluid was turbid. We also examined the onset of abdominal pain, fever (body temperature ≥ 38 °C), and bacteremia within 1 week of endoscopic procedures. Death from any cause within 1 month after the procedure was recorded.

### Statistical analysis

The baseline patient characteristics were summarized using descriptive statistics. The continuous data are presented as mean (SD) or median (interquartile range) and the categorical data as quantities and proportions. The Wilcoxon rank-sum test was used to assess the differences between the 2 groups. The comparison of categorical variables was performed using the χ^2^ test or Fisher exact test. Univariate and multivariate logistic regression analyses were performed to assess the association between patient- and procedure-related factors with peritonitis. Statistical analysis was performed using SAS 9.1 software (SAS Institute, Cary, NC).

## RESULTS

### Patient characteristics

We identified 570 PD patients who underwent 1,316 endoscopic procedures during the study period. Overall, there were 563 in the EGD group, 430 in the CS group, and 57 in the Sig group. The CS group consisted of 169 colonoscopies and 261 colonoscopies with EGD. The Sig group consisted of 52 sigmoidoscopies and 5 sigmoidoscopies with EGD. The median patient age was 56 years (range, 19–86), and males comprised 54.5% of the patients (n = 572). The mean dialysis duration was 45.5 months (range, 0–307), and 35.8% of the patients had a history of peritonitis. The other characteristics of the patients are shown in Table 1. Peritonitis developed in 3.0% of the patients after endoscopic procedures. The patients with peritonitis were older (66 vs 56 years, *P* < 0.01) with a more frequent history of peritonitis (67.7% vs 34.8%, *P* < 0.01). The dialysis duration was also significantly longer (68 vs 45 months, *P* = 0.046), and the potassium levels were lower (3.9 mEq/L vs 4.3 mEq/L, *P* = 0.012) in patients with peritonitis. There was no difference in the indication of EGD and CS for the peritonitis and nonperitonitis group (see Supplementary Table 1, Supplementary Digital Content 1, http://links.lww.com/CTG/A650).

**Table 1.** Demographics of patients with peritoneal dialysis who underwent endoscopic procedures

	Total (N = 1,050)	Peritonitis		*P*^a^
		No (n = 1,019)	Yes (n = 31)	
Age	56 (19–86)	56 (19–86)	66 (41–76)	0.002
Male	572 (54.5)	558 (54.8)	14 (45.2)	0.292
Dialysis duration (mo)	45.5 (0–307)	45 (0–307)	68 (1–279)	0.046
History of peritonitis	376 (35.8)	355 (34.8)	21 (67.7)	0.000
Use of PPIs	175 (16.7)	167 (16.4)	8 (25.8)	0.191
WBC counts (μL)	7,140 (265–35,330)	7,110 (265–35,330)	8,160 (4,380–24,180)	0.058
BUN (g/dL)	54.7 (41.2–70.9)	54.7(7.5–189.1)	54.8 (14.6–91.5)	0.721
Albumin (g/dL)	3.4 (1.7–5.1)	3.4 (1.7–5.1)	3.1 (2.0–4.6)	0.164
Creatinine (mg/dL)	10.2 (0.6–21.4)	10.2 (0.6–21.4)	8.2 (4.0–21.0)	0.066
Potassium (mEq/L)	4.2 (1.2–7.3)	4.3 (1.2–7.3)	3.9 (2.8–4.9)	0.012

Values are n (interquartile range) or n (%).

BUN, blood urea nitrogen; PPIs, proton-pump inhibitors; WBC, white blood cell.

a Wilcoxon rank-sum test.

### Clinical outcomes

Antibiotics were not administered in 716 patients, and 154 patients received prophylactic antibiotics before the endoscopic procedures (Table 2). The prior antibiotic therapy group consisted of 180 patients. The detailed indication and specific type of antibiotics are described in Supplementary Digital Content 1 (see Supplementary Table 2, http://links.lww.com/CTG/A650). Peritonitis developed in 2.0% of the patients in the nonprophylactic group (Table 2) and in 5.1% of the patients who received antibiotics. Specifically, peritonitis developed in 8.3% of the patients in the prior antibiotic therapy group and 1.3% in the prophylactic group. The prior antibiotic therapy group had an increased risk of peritonitis compared with the nonprophylactic group (odds ratio [OR] = 4.56; 95% confidence interval [CI]: 2.16–9.63; *P* < 0.01). The risk of peritonitis was not significantly different between the prophylactic group and the nonprophylactic group (OR = 0.66; 95% CI: 0.15–2.93; *P* = 0.59). The rate of abdominal pain, fever, and 30-day mortality in the prior antibiotic therapy group was 20.6%, 10.0%, and 8.9%, respectively. The corresponding rates in the nonprophylactic and prophylactic groups were 4.7%, 1.7%, 0.3% and 5.8%, 1.9%, 0.6%, respectively. These outcomes were significantly higher in the prior antibiotic therapy group (*P* < 0.05). Meanwhile, there was no significant difference in these outcomes between the nonprophylactic and the prophylactic group. The cause of death for the PD patients who received endoscopy is described in Supplementary Digital Content 1 (see Supplementary Table 3, http://links.lww.com/CTG/A650).

**Table 2.** Clinical outcomes according to use of antibiotics

	Nonprophylactic (n = 716)	Prophylactic antibiotics (n = 154)	Prior antibiotic therapy (n = 180)	*P*
Peritonitis (n = 31)	14 (2.0)	2 (1.3)	15 (8.3)^a,b^	0.005
Abdominal pain (n = 80)	34 (4.7)	9 (5.8)	37 (20.6)^a,b^	0.037
Fever (n = 33)	12 (1.7)	3 (1.9)	18 (10.0)^a,b^	0.000
Bacteremia (n = 3)	1 (0.1)	1 (0.6)	1 (0.5)	0.256
30-d mortality (n = 19)	2 (0.3)	1 (0.6)	16 (8.9)^a,b^	0.004

Values are n (%), unless otherwise defined.

a *P* < 0.05 for prior antibiotic therapy vs nonprophylactic.

b *P* < 0.05 for prior antibiotic therapy vs prophylactic antibiotics.

### Peritonitis according to endoscopic procedures

Peritonitis was observed in 1.8%, 4.2%, and 5.3% of the patients in the EGD, CS, and Sig groups, respectively (Table 3). The CS group had a higher risk of peritonitis compared with the EGD group (OR = 2.42; 95% CI: 1.10–5.29; *P* = 0.03). The Sig group showed a higher incidence of peritonitis than the EGD group but without statistical significance (OR = 3.07; 95% CI: 0.82–11.50; *P* = 0.10). In the EGD and Sig groups, biopsy and therapeutic procedures were not associated with an increased risk of developing peritonitis. Therapeutic procedures showed a significantly higher incidence of peritonitis compared with diagnostic procedures in the CS group (OR = 6.50; 95% CI: 1.63–25.88; *P* = 0.01). However, biopsies did not increase the peritonitis risk compared with the diagnostic group (OR = 2.91; 95% CI: 0.76–11.14; *P* = 0.119).

**Table 3.** Development of peritonitis after endoscopy according to the procedure invasiveness

	No peritonitis (n = 1,019)	Peritonitis (n = 31)	OR (95% CI)	*P*
EGD (n = 563)				
Diagnostic (n = 291)	286 (98.3%)	5 (1.7%)	Reference	
Biopsy (n = 245)	240 (98.0%)	5 (2.0%)	1.19 (0.341–4.165)	0.78
Therapeutic (n = 27)	27 (100.0%)	0 (0.0%)	0.00	1.00
Polypectomy (n = 9)	9 (100.0%)	0 (0.0%)	0.00	1.00
Hemostasis (n = 18)	18 (100.0%)	0 (0.0%)	0.00	1.00
CS (n = 430)				
Diagnostic (n = 184)	181 (98.4%)	3 (1.6%)	Reference	
Biopsy (n = 174)	166 (95.4%)	8 (4.6%)	2.91 (0.76–11.14)	0.119
Therapeutic (n = 72)	65 (90.3%)	7 (9.7%)	6.50 (1.63–25.88)	0.01
Polypectomy (n = 71)	64 (90.1%)	7 (9.9%)	6.60 (1.66–26.23)	0.01
Hemostasis (n = 1)	1 (100.0%)	0 (0.0%)	0.00	1.00
Sigmoidoscopy (n = 57)				
Diagnostic (n = 28)	27 (96.4%)	1 (3.6%)	Reference	
Biopsy (n = 25)	23 (92.0%)	2 (8.0%)	2.35 (0.20–27.59)	0.50
Therapeutic (n = 4)	4 (100.0%)	0 (0.0%)	0.00	1.00
Hemostasis (n = 4)	4 (100.0%)	0 (0.0%)	0.00	1.00

Values are n (%), unless otherwise defined.

CI, confidence interval; CS, colonoscopy with or without EGD; EGD, esophagogastroduodenoscopy; OR, odds ratio.

### Factors associated with peritonitis

In the univariate analysis, age, history of peritonitis, CS, therapeutic procedures, and prior antibiotic therapy increased the risk of peritonitis (Table 4). Multivariate analysis revealed that age (OR = 2.63; 95% CI: 1.18–5.90, *P* = 0.02), history of peritonitis (OR = 2.68; 95% CI: 1.19–5.06, *P* = 0.02), CS group (OR = 3.84; 95% CI: 1.67–8.84, *P* = 0.00), and prior antibiotic therapy (OR = 3.53; 95% CI: 1.58–7.88, *P* = 0.00) were significantly associated with an increased risk of peritonitis.

**Table 4.** Endoscopy-related factors and antibiotic use as risk factors for peritonitis after endoscopy in PD patients

Characteristics	Univariate OR (95% CI)	*P*	Multivariate OR (95% CI)	*P*
Male	1.52 (0.72–3.21)	0.28		
Age <60 y ≥60 y	Reference 2.58 (1.14–5.88)	0.02	Reference 2.63 (1.18–2.90)	0.02
Dialysis duration <60 mo ≥60 mo	1.03 (0.11–9.21)	0.96		
History of peritonitis	2.58 (1.13–5.86)	0.02	2.68 (1.19–5.06)	0.02
Potassium (mEq/L) ≥4 <4	Reference 0.83 (0.39–1.77)	0.63		
Type of endoscopy EGD CS Sig	Reference 4.20 (1.79–9.84) 1.18 (0.30–4.69)	0.00 0.82	Reference 3.84 (1.67–8.84)	0.00
Invasiveness of the procedure Diagnostic Biopsy Therapeutic	Reference 2.38 (0.40–14.11) 2.80 (1.18–7.68)	0.34 0.02		
Antibiotic use Nonprophylactic Prior antibiotic therapy Prophylactic	Reference 3.46 (1.52–7.88) 0.41 (0.09–1.88)	0.00 0.25	Reference 3.53 (1.58–7.88)	0.00

CI, confidence interval; CS, colonoscopy with or without EGD; EGD, esophagogastroduodenoscopy; OR, odds ratio; PD, peritoneal dialysis; Sig, sigmoidoscopy with or without EGD.

### Bacterial culture after endoscopy

Culture-negative results were reported in 16 (52%) of the patients with peritonitis. *Escherichia coli* was the main causative organism (10 cases) among the 15 culture-positive peritonitis cases. Other causative organisms included *Pseudomonas*, *Staphylococcus*, *Morganella*, *Corynebacterium*, and *Enterococcus*. Three patients developed bacteremia after their endoscopic procedure. The causative organisms of the bacteremia were *Burkholderia*, *Staphylococcus*, and *Enterococcus*.

## DISCUSSION

In our study of 1,316 endoscopies in 570 PD patients, we found that the rate of peritonitis was 3.0% within 1 week of the procedure. To our knowledge, this is the largest study to evaluate the risk of peritonitis after endoscopy in PD patients. Age, previous history of peritonitis, CS, and prior antibiotic therapy were associated with the development of peritonitis. Prior antibiotic therapy was also associated with an increased risk of abdominal pain, fever, and 30-day mortality. However, prophylactic antibiotics did not reduce the development of peritonitis. Therapeutic CS such as polypectomy increased the risk of peritonitis compared with diagnostic. Biopsy during CS did not increase the risk of peritonitis.

Peritonitis is a common complication in PD patients, with 3.5%–10.0% morbidity and mortality (1). Peritonitis is also a leading cause of patient transfer to hemodialysis, which, in turn, leads to increased costs and decreases the quality of life for patients (12,13). The prevalence of GI disorders is high in PD patients, and endoscopic evaluation is frequently needed (14). During an endoscopic investigation, both the internal and external surfaces of endoscopes are exposed to body fluids and potential contaminants. This may lead to the transmission of microorganisms from patient to patient or from the gut lumen through the bloodstream to susceptible organs (15).

The risk of infections associated with endoscopy is generally considered to be low (16). A recent nationwide study reported the 30-day infection rates of CS and sigmoidoscopy to be 0.37%, with no increased risks associated with polypectomy or biopsy (17). This study reported age and comorbidities, such as liver disease, cholangitis, cholecystitis, and chronic renal failure, as factors associated with infection. Another study reported the postendoscopic infection rates as more than 1 of 1,000 screening CS procedures and the rates for EGD were higher, at more than 3 of 1,000 (18). However, endoscopy has been associated with peritonitis development in PD patients through hematogenous, transmural, and ascending routes (2). Antibiotic prophylaxis is generally not recommended before an EGD or CS. However, recent guidelines recommend the use of intravenous or intraperitoneal antibiotics before endoscopic procedures in PD patients to lower the risk of peritonitis (2,7). This recommendation is based on retrospective observational studies that reported that antibiotic prophylaxis reduced the risk of peritonitis in patients receiving colonoscopies (9).

The current literature reports conflicting results on the role of antibiotic prophylaxis and the invasiveness of endoscopic procedures as risk factors for peritonitis (8–11). In an earlier study of 97 colonoscopies, peritonitis did not develop in patients who receive antibiotic prophylaxis (8). Biopsy and polypectomy did not increase the risk of peritonitis in this study. Another study of 125 endoscopies reported antibiotic prophylaxis significantly reduced endoscopy-associated peritonitis (11). Invasive procedures such as biopsy and polypectomy were associated with an increased risk of peritonitis. Recently, a randomized controlled trial of 93 patients reported that antibiotic prophylaxis did not reduce the development of peritonitis after colon polypectomies in PD patients (8).

The incidence of peritonitis in our study was lower than the 6%–8.5% reported in previous studies (8,9,11). The lower rate of peritonitis in our study may be due to the large number of EGDs included in our study compared with the previous studies. However, the peritonitis rate of 4.2% in the CS group was also lower than that of previous studies. CS was associated with an increased risk of peritonitis compared with EGD, and polypectomy was associated with an increased risk compared with diagnostic colonoscopies. Contrary to previous studies, biopsy during a CS was not associated with an increased risk of peritonitis. In our study, prophylactic antibiotics were not associated with a decreased risk of peritonitis. Patients in the prior antibiotic therapy group were at an increased risk of developing peritonitis compared with the nonprophylactic and prophylactic groups. These patients also showed increased risks of developing abdominal pain, fever, bacteremia, and 30-day mortality compared with the nonprophylactic and prophylactic groups. These findings have implications regarding endoscopic procedures in PD patients being treated for infections. The timing of endoscopy should be carefully considered in PD patients receiving antibiotics for infections because these patients are at an increased risk of developing peritonitis and subsequent mortality.

Recent guidelines recommend antibiotic prophylaxis before endoscopy of the lower GI tract in patients undergoing PD (7). Antibiotic prophylaxis is generally not recommended for endoscopy of the upper GI tract. Our study reported a higher rate of peritonitis in patients receiving CS compared with EGD. However, the rates of peritonitis after endoscopic procedures were not as high as previously reported even for endoscopies of the lower GI tract. Although CS with polypectomy may increase the risk of PD peritonitis, the routine administration of prophylactic antibiotics seems to have limited benefits because of the low rates of peritonitis. Our findings are consistent with 2 recent studies of 51 and 97 colonoscopies that reported no incidence of peritonitis after CS despite the lack of antibiotic prophylaxis (19,20). Our study suggests that prophylactic antibiotics should be reserved for high-risk patients (i.e., older age and history of peritonitis).

There were several limitations to our study. First, this was a retrospective study, and we cannot be sure of the causal relationship and biological plausibility of endoscopy and its association with peritonitis. Although our study included the largest number of patients to date, the small number of PD peritonitis cases recorded may have prevented us from detecting the effects of prophylactic antibiotics. Also, there were differences in the baseline characteristics between the patients who developed peritonitis and those who did not. Third, we included patients with infections that occurred within 7 days after endoscopy. Focusing on infections within 7 days may have maximized the specificity of our study, but infections with a longer incubation period may have been underestimated.

In conclusion, EGD is relatively safe to perform in PD patients. We demonstrated that PD patients with infections are at an increased risk of developing peritonitis after endoscopy. Antibiotic prophylaxis seems to be of limited value because of the low rate of peritonitis after an endoscopy but should be considered in high-risk patients. Future randomized controlled studies with a sufficient number of patients in a high-risk population are needed to better guide the management of the risk and benefits of antibiotic prophylaxis in PD patients.

## CONFLICTS OF INTEREST

**Guarantor of the article:** Jae Myung Park, MD, PhD.

**Specific author contributions:** Joon Sung Kim, MD, PhD, and Eunha Jung, MD, contributed equally to this work. J.S.K. and J.M.P.: planned the study. E.J., J.S.K., and J.M.P.: wrote the manuscript. All authors interpreted the date, revised the manuscript for intellectual content, and approved the final manuscript.

**Financial support:** This research was supported by the Basic Science Research Program through the National Research Foundation of Korea funded by the Ministry of Education, Science, and Technology (2019R1A5A2027588) and by the 2018 Research Fund from the Korean College of *Helicobacter* and Upper Gastrointestinal Research.

**Potential competing interests:** None to report.

**Study Highlights**

**WHAT IS KNOWN**

✓ Endoscopy is associated with the development of peritonitis in peritoneal dialysis (PD) patients. The role of prophylactic antibiotics for preventing peritonitis is controversial.

**WHAT IS NEW HERE**

✓ PD patients receiving antibiotics for infections before endoscopy were at a high risk of developing peritonitis. PD patients receiving therapeutic colonoscopy such as polypectomy were at a high risk of developing peritonitis.

**TRANSLATIONAL IMPACT**

✓ Prophylactic antibiotics should be reserved for patients with a high risk of developing peritonitis.

## Supplementary Material

SUPPLEMENTARY MATERIAL
