# Supplementary material for: Safety of Endoscopy in Peritoneal Dialysis Patients
Source: Clin Transl Gastroenterol. 2021 Jul 1;12(7):e00379. doi: 10.14309/ctg.0000000000000379 (PMC8345910; doi:10.14309/ctg.0000000000000379)
Supplement: SUPPLEMENTARY MATERIAL [file ct9-12-e00379-s001.docx]

|  |
| --- |

**Supplementary Table 1. Indications of patients receiving each endoscopic procedure.**

|  | Total (N=1050) | No peritonitis (n=1019) | Peritonitis (n=31) | *P** |
| --- | --- | --- | --- | --- |
| EGD  Screening  Surveillance  Functional disorders  Others | 214 (38.0)  11 (2.0)  234 (41.6)  104 (18.5) | 211 (38.2)  10 (1.8)  230 (41.6)  102 (18.4) | 3 (30)  1 (10)  4 (40)  2 (20) | 0.305 |
| CS  Screening  Surveillance  Functional disorders  Others | 310 (72.1)  18 (4.2)  63 (14.7)  39 (9.1) | 301 (73.1)  17 (4.1)  59 (14.3)  35 (8.5) | 9 (50)  1 (5.6)  4 (22.2)  4 (22.2) | 0.073 |
| Sig  Screening  Surveillance  Functional disorders  Others | 3 (5.3)  5 (8.8)  30 (52.6)  19 (33.3) | 3 (5.6)  5 (9.3)  28 (51.9)  18 (33.3) | 0  0  2 (66.7)  1 (33.3) | 0.0204 |

CS, colonoscopy with or without EGD; EGD, esophagogastroduodenoscopy; Sig, sigmoidoscopy with or without EGD.

Values are n (%) unless otherwise defined.

*Fischer’s exact test.

**Supplementary Table 2. Specific indication and type of antibiotics used in the prior antibiotic therapy group.**

| Indication | Number |
| --- | --- |
| Bacteremia  Dental procedures  Enterocolitis  Fever of unknown origin  Pneumonia  Soft tissue infection  Upper respiratory tract infection  Others | 3  12  23  4  70  37  18  13 |
| Type of antibiotics |  |
| Amoxcillin/clavulanate  Ampicillin/sulbactam  Carbapenem  Cephalosporin  Metronidazole  Piperacillin/tazobactam  Quinolone  Vancomycin  Others | 7  9  6  65  14  14  12  22  31 |

**Supplementary Table 3. Summary of PD patients who died after endoscopy.**

| Case | Sex | Age | Type of procedure | Indication | Endoscopic procedure | Peritonitis | Cause of death |
| --- | --- | --- | --- | --- | --- | --- | --- |
| 1 | 1 | 76 | EGD+CS | Screening | None | No | Septic shock |
| 2 | 1 | 80 | EGD | Bleeding | Hemostasis | No | GI bleeding |
| 3 | 1 | 67 | CS | Functional | Polypectomy | No | PD peritonitis |
| 4 | 1 | 66 | EGD | Bleeding | Hemostasis | No | GI bleeding |
| 5 | 1 | 73 | EGD | Bleeding | Hemostasis | Yes | GI bleeding |
| 6 | 2 | 67 | EGD+CS | Functional | Biopsy | No | Septic shock |
| 7 | 2 | 41 | EGD+CS | Surveillance | None | Yes | Pneumonia |
| 8 | 1 | 71 | EGD | Bleeding | None | Yes | PD peritonitis |
| 9 | 1 | 55 | CS | Surveillance | Polypectomy | No | Septic shock |
| 10 | 2 | 69 | EGD+Sig | Functional | Biopsy | Yes | Septic shock |
| 11 | 1 | 82 | EGD | Bleeding | None | Yes | Myocardial infarction |
| 12 | 1 | 62 | EGD+Sig | Screening | None | No | Septic shock |
| 13 | 1 | 68 | CS | Surveillance | Polypectomy | No | Septic shock |
| 14 | 2 | 77 | Sig | Bleeding | None | No | Respiratory failure |
| 15 | 2 | 45 | CS | Surveillance | None | No | Septic shock |
| 16 | 1 | 52 | EGD+CS | Screening | Biopsy | No | Pneumonia |
| 17 | 1 | 58 | EGD | Screening | None | No | Myocardial infarction |
| 18 | 2 | 63 | CS | Screening | Polypectomy | Yes | Pneumonia |
| 19 | 1 | 53 | EGD+CS | Screening | None | No | Myocardial infarction |

CS, colonoscopy; GI, gastrointestinal; EGD, esophagogastroduodenoscopy; PD, peritoneal daily; Sig, sigmoidoscopy.
